# Virus-induced inhibition of cardiac pacemaker channel HCN4 triggers bradycardia in human-induced stem cell system

**DOI:** 10.1007/s00018-022-04435-7

**Published:** 2022-07-21

**Authors:** Stefan Peischard, Melina Möller, Paul Disse, Huyen Tran Ho, Arie O. Verkerk, Nathalie Strutz-Seebohm, Thomas Budde, Sven G. Meuth, Patrick A. Schweizer, Silke Morris, Lena Mücher, Verónica Eisner, Dierk Thomas, Karin Klingel, Karin Busch, Guiscard Seebohm

**Affiliations:** 1 Institute for Genetics of Heart Diseases (IfGH), Department of Cardiovascular Medicine, University Hospital Münster, 48149 Münster, Germany; 2 GRK 2515, Chemical Biology of Ion Channels (Chembion), Westfälische Wilhelms-Universität Münster, Münster, Germany; 3 Department of Medical Biology, Amsterdam University Medical Centers, University of Amsterdam, 1105 Amsterdam, The Netherlands; 4 Department of Neurology, Medical Faculty, Heinrich-Heine-University, Düsseldorf, Germany; 5 Institute of Physiology I, Westfälische-Wilhems Universität Münster, 48149 Münster, Germany; 6 Department of Cardiology, Medical University Hospital Heidelberg, 69120 Heidelberg, Germany; 7 HCR (Heidelberg Center for Heart Rhythm Disorders), University Hospital Heidelberg, Im Neuenheimer Feld 410, 69120 Heidelberg, Germany; 8 DZHK (German Centre for Cardiovascular Research), Partner Site Heidelberg/Mannheim, University of Heidelberg, Im Neuenheimer Feld 410, 69120 Heidelberg, Germany; 9 Institute for Integrative Cell Biology and Physiology, Department of Biology, University of Münster, 48149 Münster, Germany; 10 Department of Cellular and Molecular Biology, School of Biological Sciences, Pontificia Universidad Católica de Chile, 8331150 Santiago, Chile; 11 Cardiopathology, Institute for Pathology and Neuropathology, University Hospital of Tuebingen, 72076 Tübingen, Germany

**Keywords:** Heart disease, Sinoatrial node, Viral replication, GTPase

## Abstract

The enterovirus Coxsackievirus B3 (CVB3) is known to be a major source for the development of cardiac dysfunctions like viral myocarditis (VMC) and dilatative cardiomyopathy (DCM), but also results in bradycardia and fatal cardiac arrest. Besides clinical reports on bradycardia and sudden cardiac death, very little is known about the influence of CVB3 on the activity of human cardiac pacemaker cells. Here, we address this issue using the first human induced pluripotent stem cell (hiPSC)-derived pacemaker-like cells, in which the expression of a transgenic non-infectious variant of CVB3 can be controlled dose- and time-dependently. We found that CVB3 drastically changed hyperpolarization-activated cyclic nucleotide-gated channel 4 (HCN4) distribution and function in hiPSC-derived pacemaker-like tissue. In addition, using HCN4 cell expression systems, we found that HCN4 currents were decreased with altered voltage dependency of activation when CVB3 was expressed. Increased autophagosome formation and autophagosomal HCN4 insertion was observed in hiPSC-derived pacemaker-like cells under CVB3 expression as well. Individual effects of single, non-structural CVB3 proteins were analyzed and demonstrated that CVB3 proteins 2C and 3A had the most robust effect on HCN4 activity. Treatment of cells with the Rab7 inhibitor CID 106770 or the CVB3-3A inhibitor GW5074 led to the recovery of the cytoplasmatic HCN4 accumulation into a healthy appearing phenotype, indicating that malfunctioning Rab7-directed autophagosome transport is involved in the disturbed, cytoplasmatic HCN4 accumulation in CVB3-expressing human pacemaker-like cells. Summarizing, the enterovirus CVB3 inhibits human cardiac pacemaker function by reducing the pacemaker channel plasma membrane density, an effect that can be corrected by pharmacological intervention of endocytic vesicle trafficking.

**Supplementary Information:**

The online version contains supplementary material available at 10.1007/s00018-022-04435-7.

## Introduction

The heart is a complex organ with a wide variety of different cells fulfilling their specific task to ensure correct generation of electrical signals and rhythmic contraction. As cells of the working myocardium, ventricular and atrial cells, generate the actual muscle force for cardiac contractility, cells of the cardiac pacemakers and conduction system generate and propagate the electrical stimuli to activate the cells of the working myocardium [9]. These cells work together to generate an active blood flow, which can be adjusted to a multitude of external conditions. Thereby, the cardiac pacemakers and conduction system is classified into several regions harboring different cell types with different tasks. The sinoatrial node (SAN) is located at the right upper chamber of the heart and represents the primary pacemaker of the heart [8]. The SAN harbors so called pacemaker cells, which generate the hyperpolarization-activated cyclic nucleotide-gated channel 4 (HCN4)-driven funny current I_f_, that leads to the controlled depolarization of pacemaker cells towards the action potential threshold [10, 35]. This electrical activity spreads via the atrial myocardium to the secondary pacemaker, the atrioventricular node (AV node). Thereafter, excitation spreads from the AV node via His bundle and purkinje network to the ventricular apex, where the ventricular contraction is triggered. Pathologic mutations, channelopathies or pathogens are known to induce dysfunctions in the working myocardium (ventricles and atria) leading to severe phenotypes, including death, in patients [2, 5, 39].

Today´s research mainly focuses on the disturbance of working myocardium function and mechanisms of cardiac dysfunction have already been identified in several cases. As one of the major pathogens causing cardiac dysfunction including bradycardia and cardiac arrest, viruses like the Coxsackievirus B3 (CVB3) have been reported [20]. Currently, pharmacological treatment to counteract enterovirus induced bradycardia and subsequent cardiac arrest is not available leading to weak prognosis of the 5-year mortality in CVB3-infected patients [12]. CVB3 has already been shown to cause myocarditis in patients and to induce apoptosis in cell culture systems [6]. Furthermore, CVB3 was shown to lead to enhanced autophagy, degradation of cellular organelles like mitochondria and elevated reactive oxygen species (ROS) production [6, 24, 36, 39]. In addition, CVB3 proteins can also have an impact on the expression, transport and plasma membrane insertion of cardiac ion channels like Kv7.1/KCNE1, potentially leading to disturbances in the generation of cardiac action potentials with possible arrhythmia [32].

The Coxsackievirus has a single-stranded RNA genome in plus-strand orientation, which is infectious itself and codes for a polyprotein. The translated polyprotein is divided into three regions (P1–P3) and is proteolytically cleaved into structural and non-structural proteins during its synthesis [13, 21]. The P1 region codes for the four structural proteins of the capsid (VP4, VP2, VP3 and VP1), the P2 region for four (2A, 2B, 2BC, 2C) and the P3 region for six non-structural proteins (3A, 3AB, 3B, 3C, 3CD, 3D). RNA processing is performed by the viral proteases 2A and 3C and the 3CD precursor protein. In addition to viral proteases, non-structural proteins also include proteins that have a pro- or even anti-apoptotic effect on the cell, increase membrane permeability and inhibit protein secretion and cellular exocytosis of the host cell [24].

Nevertheless, little is known about the modulation of ion channel trafficking in virus-infected cells, especially regarding the cardiac pacemaker system. This may be explained by the lack of sample material and uncontrolled experimental conditions during in vitro infection.

With the development of a new human induced pluripotent stem cell (hiPSC)-based cell culture system, allowing the controlled dose- and time-dependent expression of a non-infectious variant of CVB3 (CVB3∆VP0), the required test material for the investigation of enteroviral infections is available [23]. The directed differentiation of the cell line SFS.1CVB3∆VP0-IRES-Venus into ventricular-like cells and the controlled expression of CVB3∆VP0 via a doxycycline-dependent tet-on system showed transferrable phenotypes that were observed in studies performed in mice and in patients. The ROS production in the mitochondria was shown to be significantly elevated, the cardiac QT-interval was shifted and 3D holographic scans showed the degeneration of membranous structures [23]. Changes of the QT-interval [23] in ventricular-like cells and our results concerning changed Kv7.1/KCNE1 transport [32], leaves questions about the infection of SAN-like cells unaddressed. If these cardiac pacemaker cells show changes in their ion channel function, it provides an explanation for bradycardia and/or sinus arrest in viral myocarditis patients and CVB3-infected mice. Studies identified arrhythmia, heart block and conduction disturbances as common phenotype during CVB3 infections, and studies in CVB3-infected mice found conduction disturbances and sinus arrest as relevant cause for death. Ultrastructural analysis additionally proved ongoing necrosis, vacuolization and tissue degeneration in the SAN of CVB3-infected mice [14, 15, 27, 34]. Therefore, we decided to investigate the effect of CVB3 on hiPSC-derived pacemaker-like cells to elucidate mechanisms underlying lethal cardiac phenotypes in humans and mice during CVB3 infections. Here, we investigated the effect of replication-deficient CVB3 on the HCN4 channel, the main depolarizing channel driving autonomous activity of cardiac pacemaker cells. We modified an established protocol to differentiate hiPSC-derived pacemaker-like cells by Schweizer et al. [30] to generate uniform pacemaker-like cells from the SFS.1CVB3∆VP0-IRES-Venus cell line. Based on this model, we investigated the function and distribution of HCN4 under the influence of CVB3 infection mimicking an acute infection in human nodal cells, leading to conduction disturbances and even cardiac arrest.

## Materials and method

### iPSC culture

The human iPS cells, SFS.1-CVB3∆VP0-IRES-Venus, [23, 42] were incubated at 37 °C and 5% CO_2_. Cultivation was performed as described in Frank et al. [11]. In detail, the hiPSCs were cultured in the cell culture medium named FTDA medium consisting of (DMEM/F12) (Invitrogen #21331020), 5 µg/ml ITS (Becton Dickinson #354350), 0.1% human serum albumin (Biological Industries #05–720-1B), 1X CD Lipid Concentrate (Invitrogen #1905031), 1X Penicillin/Streptomycin/Glutamine (Life Technologies #10378016), 10 ng/ml FGF2 (PeproTech #100-18B), 5 ng/ml Activin A (eBioscience #34-8993-85), 0.4 µg/ml TGFβ1 (eBioscience #34-8348-82), and 50 nM Dorsomorphin (Santa Cruz #sc-200689). The medium was replaced daily. After 4 days of culture, when cells reached 100% confluence, passaging was performed by PBS washing and subsequent incubation with Accutase® solution (Sigma #A6964) in addition with 10 µM Y-27632 (AbcamBiochemicals # ab120129) for 8–10 min at 37 °C until the cells easily detached from the well bottom. The addition of FTDA medium supplemented with 10 µM Y-27632 was used to stop the Accutase® reaction. The isolated cell suspension was centrifuged for 3 min at 200×*g* and re-suspended in FTDA medium supplemented with 10 µM Y-27632. For maintenance culture, 600,000 cells were re-seeded into 1:75 diluted Matrigel (Becton Dickinson #354263) coated 6-well plates. The next day, medium change was performed with FTDA without Y-27632.

### Differentiation of SFS.1-CVB3∆VP0-IRES-VENUS into pacemaker-like cells

SFS.1-CVB3∆VP0-IRES-Venus were detached with Accutase® for 8–10 min and treated as described before. After centrifugation, D0 differentiation medium consisting of KO-DMEM (Life Technologies #10829018), 1X Penicillin/ Streptomycin/ Glutamine, 5 µg/ ml ITS, 10 µM Y-27632, 20 ng/ ml FGF2, 1 nM CHIR-99021 (Axon Medchem #Axon1386), and 0.25 to 2.0 nMBMP-4 (R&D # 314-BP-010) was used to re-suspend the cells. The concentration of BMP-4 had to be tested regularly to ensure optimal differentiation. The re-suspended cells were seeded on Matrigel coated wells at 550,000 cells/ 24-well. Medium change was performed after 24 h. The D0 medium was changed to TS-ASC medium (KO-DMEM, 5.5 mg/ L Transferrin (Sigma #T8158-100MG), 6.75 µg/L Selenium (Sigma # S5261-10G), 1X Penicillin/ Streptomycin/ Glutamine, 250 µM ascorbate (Sigma # 49,752-10G). The incubation of the cells in TS-ASC lasted for 24 h after which the medium was changed to TS-ASC + 0.5 mM C59 (Tocris #5148). The cells were kept in TS-ASC + C59 for 48 h with a medium change after 24 h. Afterwards, the cells were kept in TS-ASC medium for further 5 days until autonomous beating of the cells was observed. The beating cells were washed with PBS and incubated with TrypLE Select (1X) (Life Technologies # 12563011) supplemented with 10 µM Y-27632 for 10 min at 37 °C. After isolation and centrifugation for 3 min at 200 g, the cells were re-suspended in KO-THAI medium (KO-DMEM, 1X Penicillin/ Streptomycin/ Glutamine, 0.2% human serum albumin, 250 µM ascorbate, 5 µg/ml ITS, and 0.004% (v/v) Thioglycerol + 10 µM Y-27632 and seeded at a 1:5–1:6 ratio on 24-wells, coated with 1:75 diluted Matrigel and 0.2% gelatin in a ratio of 1:1. The next day, medium was changed to KO-THAI medium without Y-27632 [25, 43]. For pacemaker specification, medium was changed to pacemaker medium after 24 h consisting of KO-DMEM, 20% FBS superior (Sigma Aldrich #S0615) and 1 mM CaCl_2_, which is an adaptation of the maturation medium of Schweizer et al. 2017 [30]. The young hiPSC-derived cardiomyocytes were cultivated in pacemaker medium for another 6 weeks until reaching adequate maturity. For ventricular specification, cells were cultivated for 6 weeks in KO-THAI medium with medium changes every second day.

### Immunofluorescence staining and imaging

Cell fixation was performed with an incubation with 4% PFA/PBS for 15 min. Afterwards the fixed cells were washed with PBS-T. The blocking was done with 2% BSA, 2% glycine, 0.2% Triton-X in PBS-Tween (PBS-T) for 1 h. After a washing step with PBS-T, the cells were rinsed and incubated with the primary antibodies against N-Cadherin (ab22744; Abcam), HCN4 (APC-052; Alomone Labs), Nav1.5 (ASC-013; Alomone Labs), Cav1.2 (ACC-003; Alomone Labs) Rab7 (R8779, Merck) and/or LC3 (NB100-2220SS, Novusbiologicals) at 4 °C, overnight in 0.5% BSA, PBS-T in a ratio 1:500. The next day, cells were washed with PBS-T three times and incubated with the secondary antibodies (115-585-044; Jackson Immuno Research, SAB4600036; Sigma Aldrich or A-11046; Thermo Fisher) in 0.5% BSA, PBS-T in a ratio of 1:1000. Alternatively, the cells were incubated with the conjugated antibodies for autophagy markers LC3 (NB100-2220AF350; Novus Biologicals), Beclin-1 (NB500-249AF350, Novus Biologicals) or p62 (NBP1-48320AF350 Novus Biologicals). The stained cells were fixed on glass with Aquapolymount® (18606; Polysciences) and imaged with a DMI4000 confocal microscope (Leica) equipped with a 63 × oil immersion objective or a TCS SP8 SMD cLSM (Leica) equipped with an 63 × water objective. Quantification of fluorescence signals was performed on non-edited pictures taken under identical conditions to ensure data validity.

### FACS analysis

Differentiated ventricular-like and pacemaker-like cells were washed with PBS and detached under the use of 1 ml of TrypLE Select 10× (Life TechnoligiesA1217701) for 10 min. The TrypLE reaction was stopped with 1 ml of Medium and cells were centrifuged for 2 min at 200×*g*. The cell pellet was re-suspended in PBS together with 1:500 Antibodies for connexin 43, connexin 40 and connexin 45 (sc-271837 AF647; sc-374354 AF488; sc-365107 AF594; Santa Cruz). The Antibody was incubated for 15 min at 37 °C. The cells were then centrifuged another time at 200×*g* for 2 min. The supernatant was removed carefully and the pellet was washed once with PBS. The cells were re-suspended in 500 μL PBS and analyzed with the FACSAria III Cell Sorter (BDBioscences). Data analysis was performed via FACSDiva Software Version 6.1.3 (BDBiosciences). Antibody signals were compared to unstained samples of the same differentiation for background evaluation.

### Contraction analysis

hiPSC-derived ventricular-like cells and pacemaker-like cells were directly transferred from the incubator, 37 °C and 5% CO_2_, to the microscopy setup. Video recording was performed with an OrcaFlash4.0 camera at 30 Hz. Contraction analysis was performed with Musclemotion in ImageJ.

### Multielectrode array

Five days prior measurement, the pacemaker-like cells were seeded onto Matrigel + 0.2% gelatin coated 256-9wellMEA300/30iR-ITO-mq MEA chips (Multichannel systems) in a density of 100,000 cells/well and either kept in pacemaker medium or pacemaker medium supplemented with 2 μg/ml doxycycline to start the expression of CVB3∆VP0. The medium was changed every second day. The seeded MEA chips were kept at 37 °C and 5% CO_2_. On the day of measurement, the MEA chips were put into the USB-MEA256-System (Multichannel Systems). Prior to measurement, a viability test of the Chip was run to verify the functionality of the electrodes in each of the 9 wells and to monitor the background noise. Subsequently, the actual measurement was undergone at room temperature. First, the cells were measured under basal conditions in pacemaker medium to monitor spontaneous electrical activity of the pacemaker-like cells. This was done for 10 min. After the basal measurement, ivabradine in a final concentration of 10 μM was carefully applied to each well. The wells were measured for another 10 min. The data were acquired via Cardio2D+ and analyzed with OriginPro. the exemplary traces were smoothened with OriginPro´s internal Savitzky-Golay filter using a 400-point filter.

### Pharmacological inhibition of CVB3 and Rab7 to reverse cytoplasmatic HCN4 accumulation in HeLa cells

Wildtype HeLa cells were seeded onto 12 mm glass coverslips in a density of 100.000 cells/24 well. The cells were kept in HeLa cell medium consisting of 10% FBS (S 0615; Biochrom), 1% Hepes (15630106; Thermo Fisher); 1% Non-Essential Amino Acids (M7145-100ML; Sigma Aldrich), 1% L-Alanyl-Glutamine (03-022-1B; Neo-Lab) and 1% Penicillin/Streptomycin (P4333-100ML; Sigma Aldrich) in DMEM. The next day, the cells were transfected with HCN4-pEGFP and with either CVB3-2C/pIRES-dsRed-Express2 or CVB3-3A/pIRES-dsRed-Express2 under the use of Lipofectamine3000 Transfection reagent (L3000001, Thermo Fisher). Pharmacological treatment was started directly after transfection. The HeLa cells were either treated with 0.1% DMSO; 2.5 μM N6-Benzyladenosine (4294-16-0; Santa Cruz); 10 μM GW5074 (1381, Tocris) or 1 μM CID 1067700 (ML282; Axonmedchem) for 8 h, 12 h or 24 h. The cells were immunostained against HCN4, Rab7 and LC3, as described before, and imaged. Colocalization analysis was performed with the JACoP plugin for ImageJ. Obtained Pearson´s coefficients (*r*), which describe the grade of localization, were multiplied with themselves to generate (*r*^2^) which was then used to give a percentual colocalization statement [26, 29].

### Heterologous expression and electrophysiological analyses in *Xenopus laevis* oocytes

Preparation of cRNAs and DNA constructs encoding human HCN4 and CVB3 constructs were done as previously described [19, 32]. Human HCN4 and viral genes were subcloned into the oocyte expression vector pSGEM. The constructs were linearized by NheI digest and cRNA was synthesized using an in vitro transcription kit mMessage mMachine T7 kit by Ambion. Electrophysiological experiments in oocytes were similar as previously reported [32]. In brief, *Xenopus laevis* oocytes (stages V and VI) were obtained by a commercial source (EcoCyte bioscience, Castrop-Rauxel, Germany). Oocytes were injected with human HCN4 cRNA (4 ng) and CVB3ΔVP0 or individual CVB3 proteins cRNA (2 ng). The oocytes were kept 18 °C in modified ND96-storage solution containing (in mM): 96 NaCl, 4 KCl, 1.8 MgCl_2_, 0.1 CaCl_2_, 5 HEPES (*N*-[2-hydroxyethyl] piperazine-*N*′-[2-ethanesulfonic acid]), gentamycin (50 mg/l); pH 7.6). Two-electrode voltage clamp (TEVC) recordings were conducted 3–4 days after injection using a Turbo Tec-10CX (NPI, Tamm, Germany) amplifier. Electrophysiological data were recorded and analyzed with GePulse and Ana (Dr. Michael Pusch, Genova, Italy; http://users.ge.ibf.cnr.it/pusch/). Data analysis was done using OriginPro 2018 (OriginLab Corporation, Northampton, MA, USA). Recording pipettes were filled with 3 M KCl and had resistances of 0.4–1.5 MΩ. Channel currents were recorded 3 days after injection at room temperature in ND96 recording solution containing (in mM): 96 NaCl, 4 KCl, 1.8 MgCl_2_, 1.0 mM CaCl_2_, and 5 mM HEPES; (pH 7.6).

HCN4 currents were activated by applying xx-s hyperpolarizing test potentials between − 140 mV and − 30 mV, starting from a holding potential of 0 mV. Peak tail current analysis at − 140 mV and after varying test pulses was used to assess the voltage dependence of the HCN4 channel activation. The peak current values of individual cells were fitted to a Boltzmann equation of the form:

$$ I_{t} = (1 - \min { - }P_{o} /\left[ {1 - \exp \left( {\left( {V - V_{1 / 2} } \right)/k} \right)} \right] - \min { - }P_{o} , $$

to obtain the voltage required for half-maximal activation (*V*_1⁄2_), slope factor (*k*), and the minimum open probability (min-*P*_o_, defined as the minimum value of relative tail current as done before [19]. The obtained data were analyzed using unpaired t test and one-way-ANOVA where indicated. Data were presented as mean values, and error bars indicated SEM. Significance levels of the test showing p values were indicated by stars as follows: ***p < 0.001, ****p < 0.0001.

## Results

### Development of human iPSC-based pacemaker aggregates with inducible expression of CVB3.

**Fig. 1:**
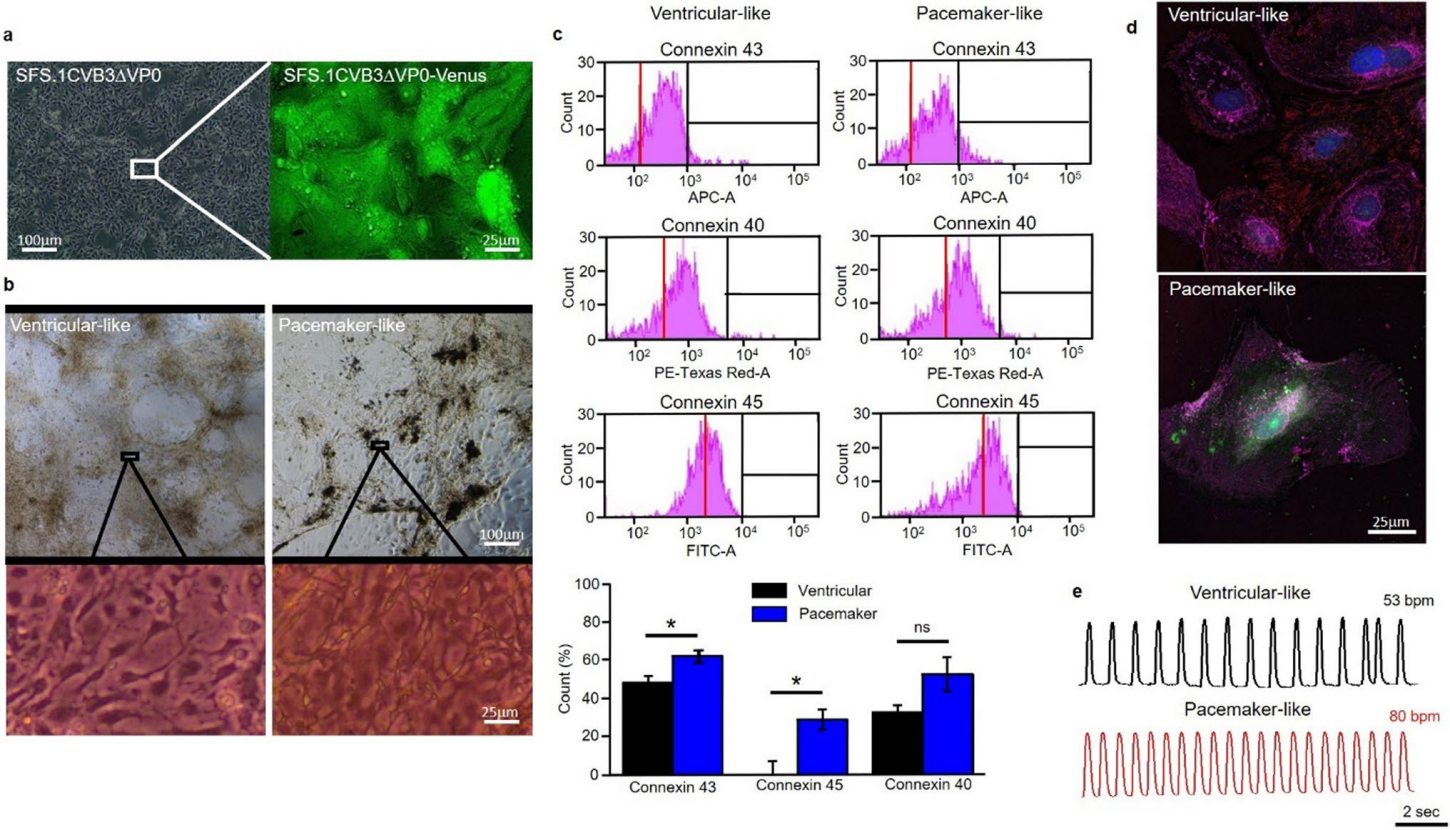
General cell culture and human-induced pluripotent stem cell (hiPSC)-derived pacemaker differentiation. **a** SFS.1CVB3∆VP0 hiPSC in culture. Left: Phase contrast image of SFS.1CVB3∆VP0 cells. Right: Fluorescence microscopy picture of doxycycline-induced SFS.1CVB3∆VP0 cells. The green VENUS signal indicates sufficient CVB3 expression. **b** Differentiated hiPSC cells in culture. Left up: Differentiated ventricular-like cells in an autonomously contracting network. Left down: Zoom into the ventricular-like cell network structure. Right up: Differentiated pacemaker-like cells. Right down: Zoom into the pacemaker-like cell network structure. **c** FACS analysis of differentiated ventricular-like cells and pacemaker-like cells showing expression of connexins 40, 43 and 45 (*n* = 3). Connexin 45 indicates sinoatrial node (SAN)-like differentiation and was only found in hiPSC-derived pacemaker-like cells. Background signal of non-stained cells of the same sample is indicated with the red line. Bottom panel: Statistical analysis shows significant increase of SAN-specific connexin 45 in hiPSC-derived pacemaker-like cells and significantly more cardiac connexin 43 (*p < 0.05). **d** Immunofluorescent antibody staining of hiPSC-derived ventricular-like cells and hiPSC-derived pacemaker-like cells against connexin 43 (magenta), connexin 40 (red) and connexin 45 (green). **e** Contraction profiles of beating ventricular-like cells and pacemaker-like cells

The novel cell line SFS.1CVB3∆VP0 hiPSC is capable of expressing the non-infectious CVB3 variant CVB3∆VP0. The viral expression is controlled via a doxycycline-dependent tet-on system. Positive CVB3 expression is indicated by Venus-marker expression (Fig. 1a). SFS.1CVB3∆VP0 was transdifferentiated into cardiac cells with either ventricular-like characteristics or cardiac pacemaker-like characteristics following a specific protocol [30]. After a differentiation-and maturation-time of about 6 weeks, the two differentiated cell types were compared. The ventricular-like cells show the typical appearance as they grow in a coarse-meshed network of cell fibers, allowing for contraction (Fig. 1b left up). Analysis of magnified pictures of the ventricular-like cells reveals their flattened and slight quadratic phenotype reminiscent of young ventricular cells in the mammal e.g. the human heart (Fig. 1b left down). In contrast, the differentiated pacemaker-like cells lack the widespread, contractile network characteristic of ventricular-like cells and grow in optically compressed clusters connected with thin, cellular fibers. A magnified view on the pacemaker-like cells reveals their distinct elongated shape clearly distinguishing these cells from the differentiated ventricular-like cells. (Fig. 1b right). For expression characterization of the two differentiated cell types, FACS analysis detecting the cardiac connexins (connexin 43, connexin 40 and connexin 45) was performed. Differentiated ventricular-like cells show strong connexins 43 and connexin 40 signals, which are mostly associated with the working myocardium. SAN-specific connexin 45 was not detected [3]. In contrast, the hiPSC-derived pacemaker-like cells showed definite and increased signals for connexin 43, connexin 40 and also connexin 45 compared to the ventricular-like cells. It is to mention, that only the signal increase for connexin 43 and connexin 45 was significant, while the signal for connexin 40 only showed a tendency for signal elevation. The significant increase of the cardiac connexin 43 additionally suggests a more complete maturation of the cells towards pacemaker-cardiac tissue in pacemaker medium compared to the medium used for maturation of ventricular-like cells (Fig. 1c). The expression and correct/typical localization of the connexins 43, 40 and 45 was controlled via immunofluorescence staining of hiPSC-derived pacemaker-like cells and ventricular-like cells with antibodies targeting the three connexins. HiPSC-derived ventricular-like cells showed strong expression of connexin 43 and connexin 40 with a well-defined membrane localization. Specific signals for connexin 45 were not observed among hiPSC-derived ventricular-like cells (Fig. 1d upper picture; Fig. 8b upper panel). HiPSC-derived pacemaker-like cells were positive for connexin 43, connexin 40 and also connexin 45. Interestingly, it was observed that the signals for connexin 43 showed almost no specific membrane localization. Connexin 43 was accumulated in direct proximity to the nucleus and in undefined cytoplasmatic areas close to the cell membrane. Furthermore, the overall signal intensity of connexin 40 seemed lower compared to signals in hiPSC-derived ventricular-like cells. In contrary, connexin 45 signals were clearly visible in hiPSC-derived pacemaker-like cells and also defined membrane localization was observed (Fig. 1d lower picture; Fig. 8b lower panel). Both cell types were also monitored to detect differences in their contraction behaviour. The analysis of the contraction behaviour of matured ventricular-like cells and pacemaker-like cells showed clear differences. The pacemaker-like cells contracted fast and with a precise rhythm whereas ventricular-like cells are slower in comparison and occasionally show single beat irregularities (Fig. 1e; Video 1, 2). Additional immunostaining of differentiated pacemaker-like cells against the cardiac-specific ion channels HCN4, Nav1.5 and Cav1.2 showed the presence of all three channels, indicating potential pacemaker function (Fig. 8a). The pacemaker cells thus exert typical key features of SAN cells that clearly differentiate them from ventricular-like cells.

### Human iPSC-based pacemaker aggregates show altered HCN4 channel localization upon CVB3 expression

**Fig. 2:**
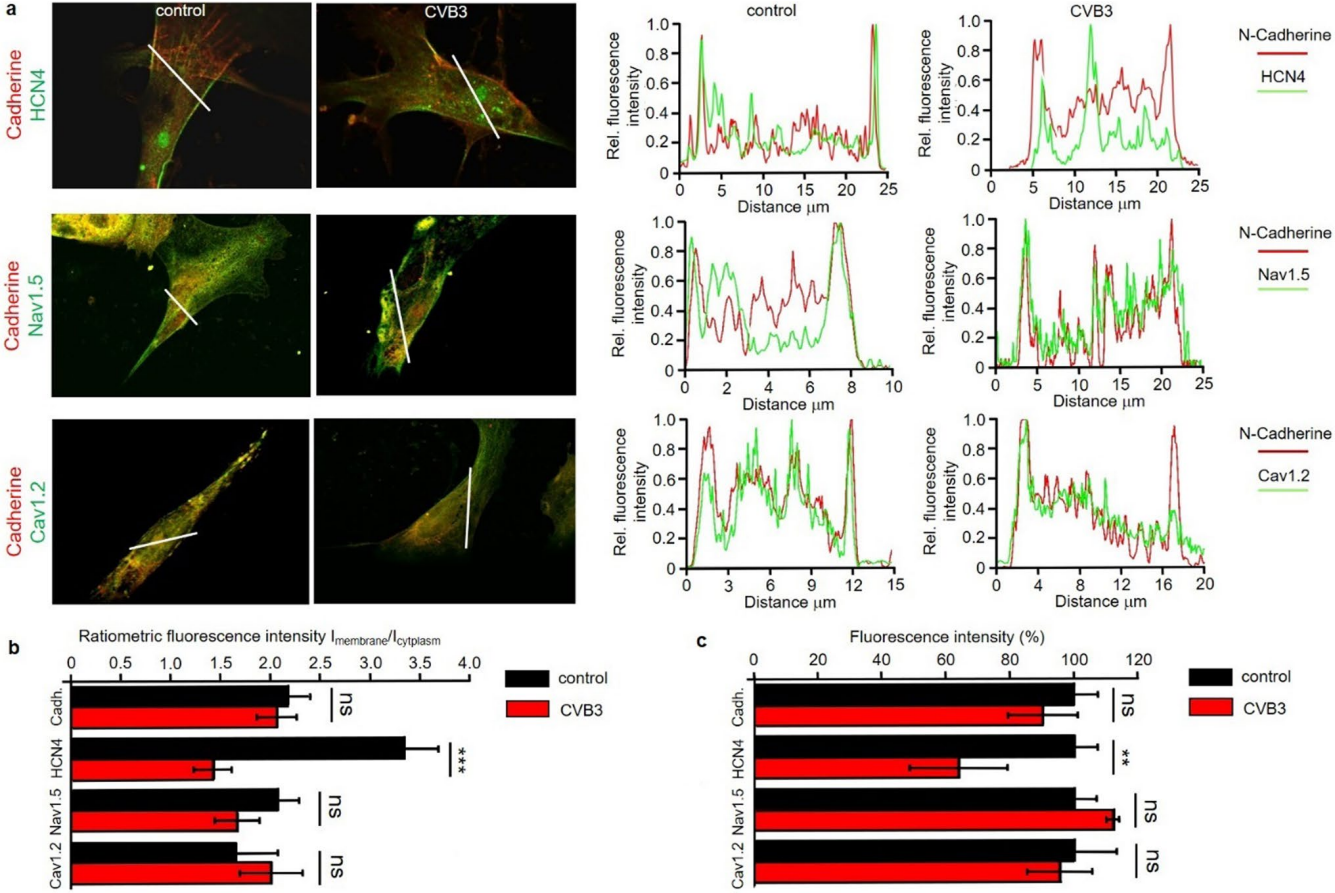
Immunofluorescence staining and analysis under basal conditions (control) and after 5 days of CVB3 expression (CVB3) in pacemaker-like cells. **a** Immunofluorescence staining of pacemaker-like cells with antibodies against N-Cadherin (membrane marker) and either anti-HCN4 (top panel), anti-Nav1.5 (middle panel) and anti-Cav1.2 (bottom panel). The distributions were visualized via linescan. **b** Quantitative distribution analysis of N-Cadherin (*n* = 30), HCN4 (*n* = 30), Nav1.5 (*n* = 5) and Cav1.2 (*n* = 5). The localization of all targets in the membrane and the cytoplasmatic fraction was monitored and put into a ratiometric dependence (***p < 0.001). **c** Quantitative expression analysis of N-Cadherin (*n* = 30), HCN4 (*n* = 30), Nav1.5 (*n* = 5) and Cav1.2 (*n* = 5). The whole fluorescence intensity of all targets in the cells was monitored and put into a percental dependence (**p < 0.01)

After initial characterization of the differentiated, pacemaker-like cells, the expression of CVB3 was induced and the investigation of viral effects on hiPSC-derived pacemaker-like cells was conducted. Immunofluorescence staining of HCN4 was performed under control conditions and after 5 days of CVB3 expression. Linescans and ratiometric analyses of pacemaker-like cells stained against HCN4 and N-Cadherin as membrane marker indicates overlapping localization and therewith a strong membrane localization of HCN4. After 5 days of CVB3 expression, HCN4 membrane abundance was reduced and cytoplasmatic accumulation of HCN4 observed (Fig. 2a, b). In contrast, Nav1.5 and Cav1.2 showed no significant cytoplasmatic accumulation upon CVB3 expression (Fig. 2a, b). Overall expression of N-Cadherin, HCN4, Nav1.5 and Cav1.2 were assessed via whole cell fluorescence intensity. Upon CVB3 expression, N-Cadherin, Nav1.5 and Cav1.2 showed no significant changes in whole-cell signal intensity compared to the control. Only the HCN4 signal declined by about 40% after 5 days of CVB3 expression (Fig. 2c).

### CVB3 alters autonomous activity by reducing HCN channels

**Fig. 3:**
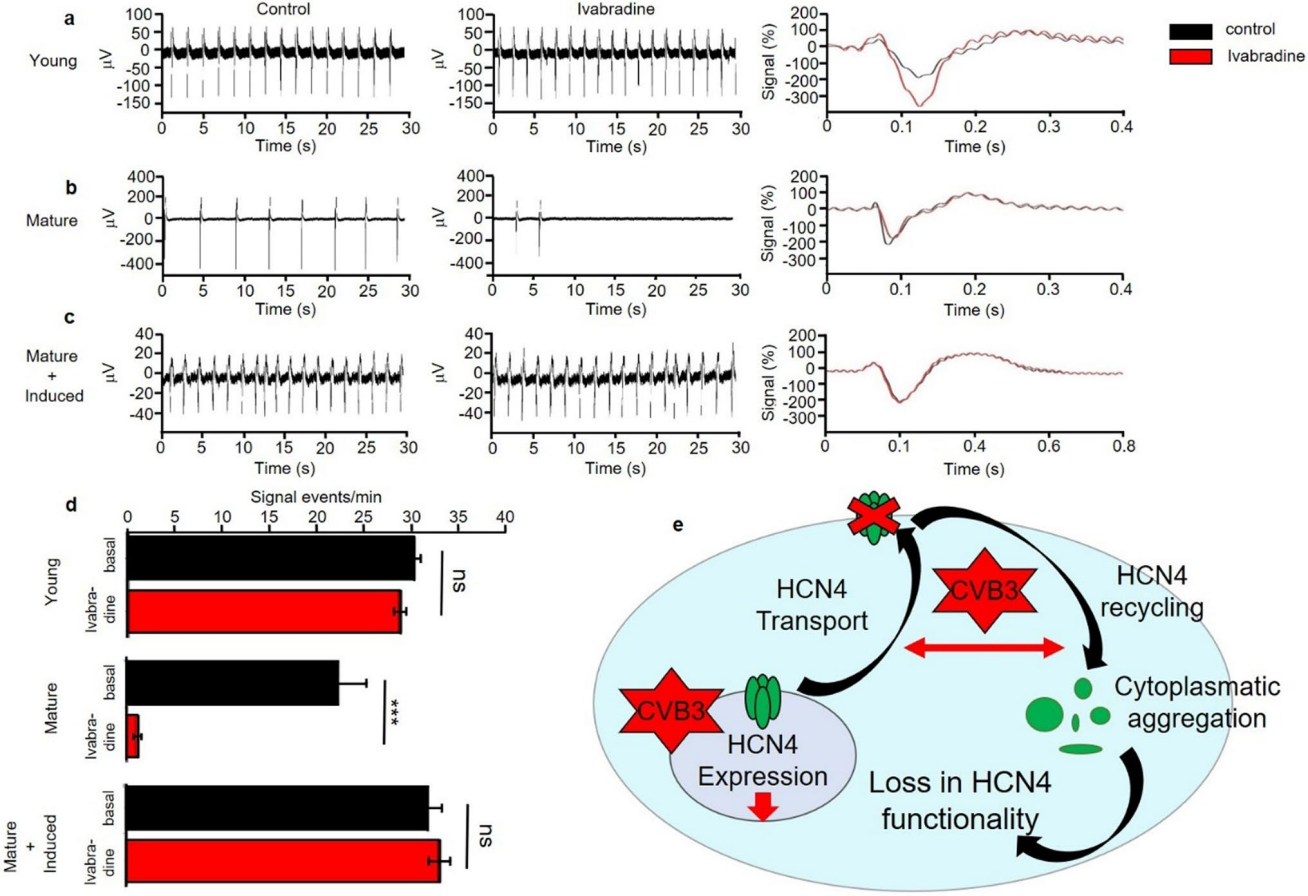
Electrophysiological analysis of differentiated pacemaker-like cells. **a** Multielectrode array (MEA) measurements of young (14 days old) pacemaker-like cells. Cells were first monitored under basal conditions (left) and then under the influence of 10 μM ivabradine (middle) for HCN4 block. The signal events were averaged (10 signals) for detailed signal analysis (right). **b** Multielectrode array of aged (40 days old) pacemaker-like cells. Cells were first monitored under basal conditions (left) and then under the influence of 10 μM ivabradine (middle) for HCN4 block. The signal events were averaged (10 signals) for detailed signal analysis (right). **c** Multielectrode array of aged (40 days old) pacemaker-like cells with additional 5 days of CVB3 induction. Cells were first monitored under basal conditions (left) and then under the influence of 10 μM ivabradine (middle) for HCN4 block. The signal events were averaged (10 signals) for detailed signal analysis (right). **d** The number of electrochemical events in the MEA measurements of young, aged and aged + CVB3 -induced pacemaker-like cells was counted under basal conditions and under the influence of 10 μM ivabradine (***p < 0.001). **e** Schematic illustration of the potential signaling pathways, which are disturbed by CVB3. Whole HCN4 expression and a transport mechanism of expressed HCN4 in the cell were found disturbed

For electrical characterization of the differentiated pacemaker-like cell aggregates, multielectrode array (MEA) measurements were undertaken. Therefore, young (14-day-old) and matured (40-day-old) pacemaker-like cells and matured, CVB3-induced (40-day-old + 5 days of CVB3 induction) pacemaker-like cells were compared under basal conditions and under 10 μM ivabradine, a potent HCN channel blocker. The whole MEA recording was undertaken at room temperature. Young pacemaker-like cells showed a mean signal generation rate of 30.30 signals/min (± 0.576 SEM) under basal conditions and 28.85 signals/min (± 0.501 SEM) after ivabradine supplementation (Fig. 3a left and middle). Statistical analysis revealed no significant differences between basal condition and ivabradine supplementation in young pacemaker-like cells (Fig. 3a left, middle and 3d) pointing to calcium-dependent autonomous activity at this stage. Averaging of ten cell signals revealed an immature shape with no defined sodium or potassium peaks and an average duration of about 400 ms (Fig. 3a right). After 6 weeks of maturation, autonomous electrical activity features more matured cardiac signal compared to the young pacemaker-like cells. Well-defined sodium and potassium potentials are visible indicating sufficient de- and repolarization phases suggesting extensive HCN4-induced autonomous activity. In accordance with this hypothesis, application of HCN4 inhibitor ivabradine changes the cell´s signal generation behaviour. While matured pacemaker-like cells showed a signal generation rate of 22.31 signals/min (± 3.00 SEM), addition of ivabradine dramatically reduced autonomous activity to a rate of only 1,14 signals/min (± 0.39 SEM). In all samples, autonomous activity finally stopped at the latest 5 min after ivabradine application (Fig. 3b left, middle and d). Consistent with the expected HCN4 function as depolarizing current *I*_f_ generator, inhibition by Ivabradine elongated the depolarization time without changing the repolarization phase. The overall signal duration lasted for about 300 ms (Fig. 3b right). 5 day-expression of CVB3 in mature pacemaker-like cells lead to an increased autonomous activity (rate of 31.88 signals/min ± 1.50 SEM), comparable to the signal generation rate of the previously described young pacemaker-like cells. Subsequent ivabradine addition did not decrease the signal generation rate, as we measured 33.18 signals/min (± 1.10 SEM). The HCN4-inhibition by ivabradine in matured, 5 days CVB3-expressing pacemaker-like cells was completely abolished (Fig. 3c left, middle and d). Signal averaging showed an elongated signal duration of about 750 ms in including a slowed rate of depolarization and an extended repolarization phase. The averaged basal signal and the averaged signal under ivabradine influence show no changes, supporting the loss of HCN4 function due to CVB3 expression (Fig. 3c right).

### CVB3 and individual non-structural CVB3 proteins reduce HCN4 function in heterologous expression

**Fig. 4:**
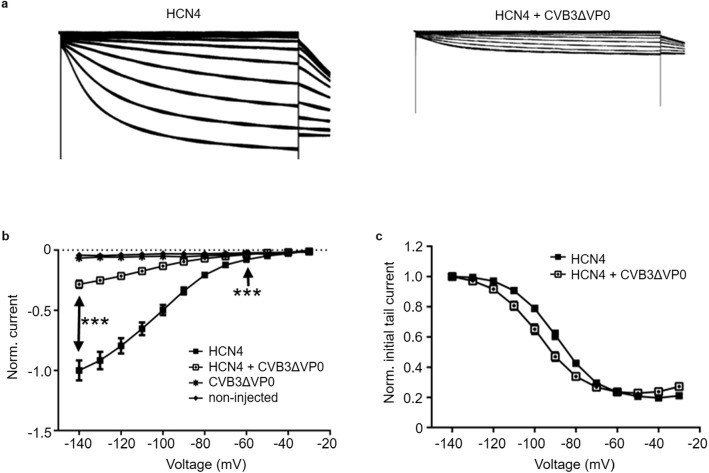
Influence of CVB3 on the channel functionality of the HCN4 Channel. Representative current traces and **a** normalized current–voltage (I–V) curves **b** of the HCN4 channel without (*n* = 32) and with coexpression of CVB3 (*n* = 35) after 5 days of channel expression. The injection of cRNA was performed separately and in each case two days before the electrophysiological recordings. The currents were activated by applying xx-s hyperpolarizing test potentials between − 140 and − 30 mV, starting from a holding potential of 0 mV. The current amplitudes of the HCN4 channel under CVB3 influence were normalized to the control current at − 140 mV of the HCN4 channel without CVB3 influence (***p < 0.001). Non-injected cells and cells with only CVB3 expression did not resulted in HCN4 currents. **c** The normalized mean initial tail currents (± SEM) of the HCN4 channel without (*n* = 32) and with coexpression with CVB3 (*n* = 35)

To study the electrophysiological properties of the HCN4 channel under the influence of CVB3, the channel was expressed alone or together with non-infectious CVB3 in *Xenopus laevis* oocytes. HCN4 cRNA was injected five days prior to the two-electrode voltage clamp recordings. The injection of CVB3 cRNA was carried out separately and in each case two days before the recordings. Characteristic HCN4 currents showed a significant reduction in coexpression with CVB3 compared to controls without CVB3 (Fig. 4a, b). To characterize the modulation of the functional properties of HCN4 by CVB3 more precisely, the influence on the voltage dependence of channel activation was investigated by classical tail current analysis (Fig. 4c). Using a Boltzmann function, the parameters V_1/2_ and k_B_ were determined for individual recordings. The results showed a clear shift of the activation curve under CVB3 influence towards more hyperpolarizing potentials. Thus, the value of the half-maximal activation *V*_1/2_ of control HCN4 channels was − 89.43 ± 1.39 mV, whereas it was significantly shifted to − 98.68 ± 1.52 mV upon coexpression with CVB3. The Boltzmann constant k_B,_ i.e., the slope of the curve, was unaffected by CVB3 (9.65 ± 0.28 mV in presence of CVB3 vs 9.58 ± 0.30 mV without CVB3), which indicates that the measured shift in *V*_1/2_ was not measured due to a change in the HCN4 channel activation state.

### Individual non-structural CVB3 proteins reduce HCN4 function

**Fig. 5:**
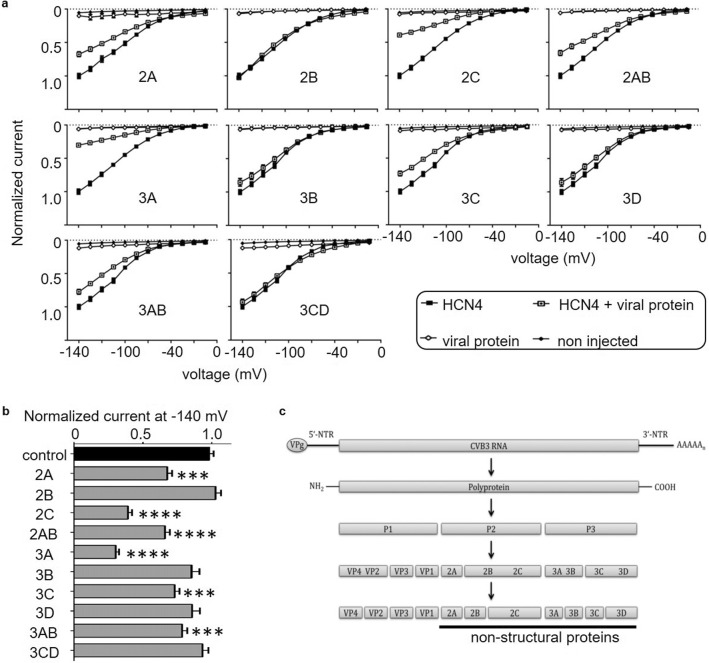
Impact of non-structural CVB3 proteins on the HCN4 channel function. **a** and **b** Normalized (−V) curves (± SEM) and the mean normalized currents at − 140 mV (***p < 0.001; ****p < 0.0001) of the HCN4 WT channel alone (*n* = 41) and with coexpression of the non-structural proteins 2A (A, *n* = 14), 2B (B, *n* = 27), 2C (C, *n* = 35), 2BC (D, *n* = 31), 3A (E, *n* = 35), 3B (F, *n* = 30), 3C (G, n = 29), 3D (H, *n* = 32), 3AB (I, *n* = 30), 3CD (J, *n* = 36) after 5-day expression of the channel. Injection of cRNA of viral proteins was performed separately and 2 days or in case of 2A 1 day before the electrophysiological measurements were performed. The currents were activated by applying xx-s hyperpolarizing test potentials between − 140 mV and − 30 mV, starting from a holding potential of 0 mV. The current amplitudes of the HCN4 channels expressed alone or in coexpression with the respective non-structural protein was analyzed at − 140 mV. **c** Genome and proteins of Coxsackievirus B3 and enteroviral polyprotein processing

Since it was shown that HCN4 coexpression with CVB3 leads to a significantly reduced current amplitude of HCN4, the individual effect of single, non-structural proteins on I_f_ were analyzed in more detail. For this purpose, the HCN4 channel was coexpressed alone or with a viral protein in *Xenopus* oocytes. Again, the individual viral proteins were injected separately, 2 days before the electrophysiological measurements were performed. After 2 days of expression of the viral protease 2A, the oocytes were too damaged to be measured. In addition, the lifespan of the oocytes could not be prolonged even by injecting smaller amounts of cRNA. Therefore, the injection of 2A was done only one day prior recording. The coexpression of HCN4 with the viral proteins 2C and 3A resulted in dramatic reduction of the HCN4 current amplitude by about 60–70%. The viral proteins 2A, 2BC, 3C and 3AB led to mild reduction of the currents between 22 and 40%. The coexpression of the viral proteins 2B, 3B, 3CD and 3D, however, led to little or no reduction (≥ 15%) of the HCN4 current amplitude (Fig. 5a, b). Because the CVB3 proteins 2C and 3A had the most robust effect on HCN4 activity in Two-Electrode-Voltage-Clamp (TEVC) recordings, those two proteins were investigated further in HeLa cells to determine cellular HCN4 localization.

### Autophagy is the potential mechanism for cytoplasmatic HCN4 accumulation

**Fig. 6:**
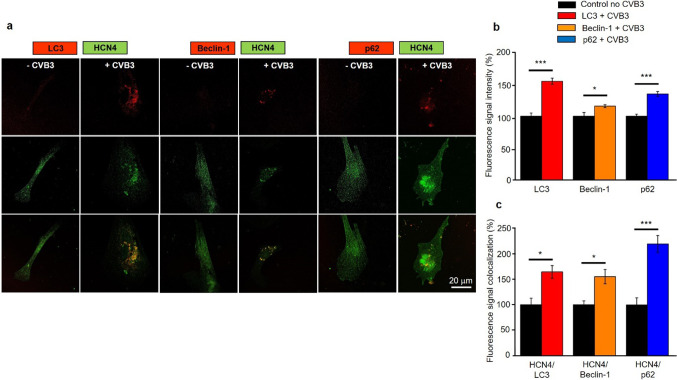
CVB3 expression leads to enhanced autophagy and autophagy-related HCN4 internalization in hiPSC-derived pacemaker-like cells. **a** Immunofluorescence staining of hiPSC-derived pacemaker-like cells without CVB3 expression and after 5 days of CVB3 expression. The cells were stained against HCN4 and the autophagy markers LC3, Beclin-1 or p62. **b** The cellular fluorescence intensities of the fluorescence markers LC3 (*n* = 13), Beclin-1 (*n* = 13) and p62 (*n* = 16) were analyzed in CVB3-expressing, hiPSC-derived pacemaker-like cells and normalized to CVB3-negative control cells (*p < 0.05; **p < 0.01; ***p < 0.001). **c** The immunostained hiPSC-derived pacemaker-like cells were analyzed for colocalization of HCN4 and the autophagy markers LC3 (*n* = 7), Beclin-1 (*n* = 11) and p62 (*n* = 15) with JAcoP in ImageJ (*p < 0.05; **p < 0.01; ***p < 0.001)

The enteroviral Proteins 2C and 3A are known to be involved in cellular autophagy induction and autophagosome formation to enable potent viral replication. As TEVC screening identified CVB3-2C and CVB3-3A as main causes for HCN4 loss-of-function, we hypothesized that CVB3-induced autophagosome formation might be the cause for HCN4 re-localization from the cell membrane to the cytoplasm. CVB3 expression in hiPSC-derived pacemaker-like cells indeed resulted in a significant increase of the autophagosomal markers LC3, Beclin-1 and p62. The LC3 signal within hiPSC-derived pacemaker-like cells increased by 54.9% (± 4.9 SEM), the Beclin-1 signal increased by 15.9% (± 2.5 SEM) and the p62 signal increased by 35,0% (± 3.8 SEM) indicating general increase of autophagosome formation during CVB3 expression (Fig. 6a, b). Optical colocalization analysis of the mentioned autophagosomal markers LC3, Beclin-1 and p62 with HCN4 was performed to investigate whether cytoplasmatic HCN4 localization and increased autophagosome formation correlated. The analysis of the colocalization of HCN4 with either LC3, Beclin-1 or p62 showed a significant increase in colocalization when CVB3 was expressed. The colocalization of HCN4 and LC3 increased by 64.4% (± 12.4 SEM), the colocalization of HCN4 with Beclin-1 was increased by 55.1% (± 13.9 SEM) and the colocalization of HCN4 and p62 increased even by 118.9% (± 11.4 SEM) clearly showing autophagosome formation and HCN4 incorporation therein (Fig. 6c).

### Identification of RABs involved in HCN4-CVB3 sensitivity

**Fig. 7:**
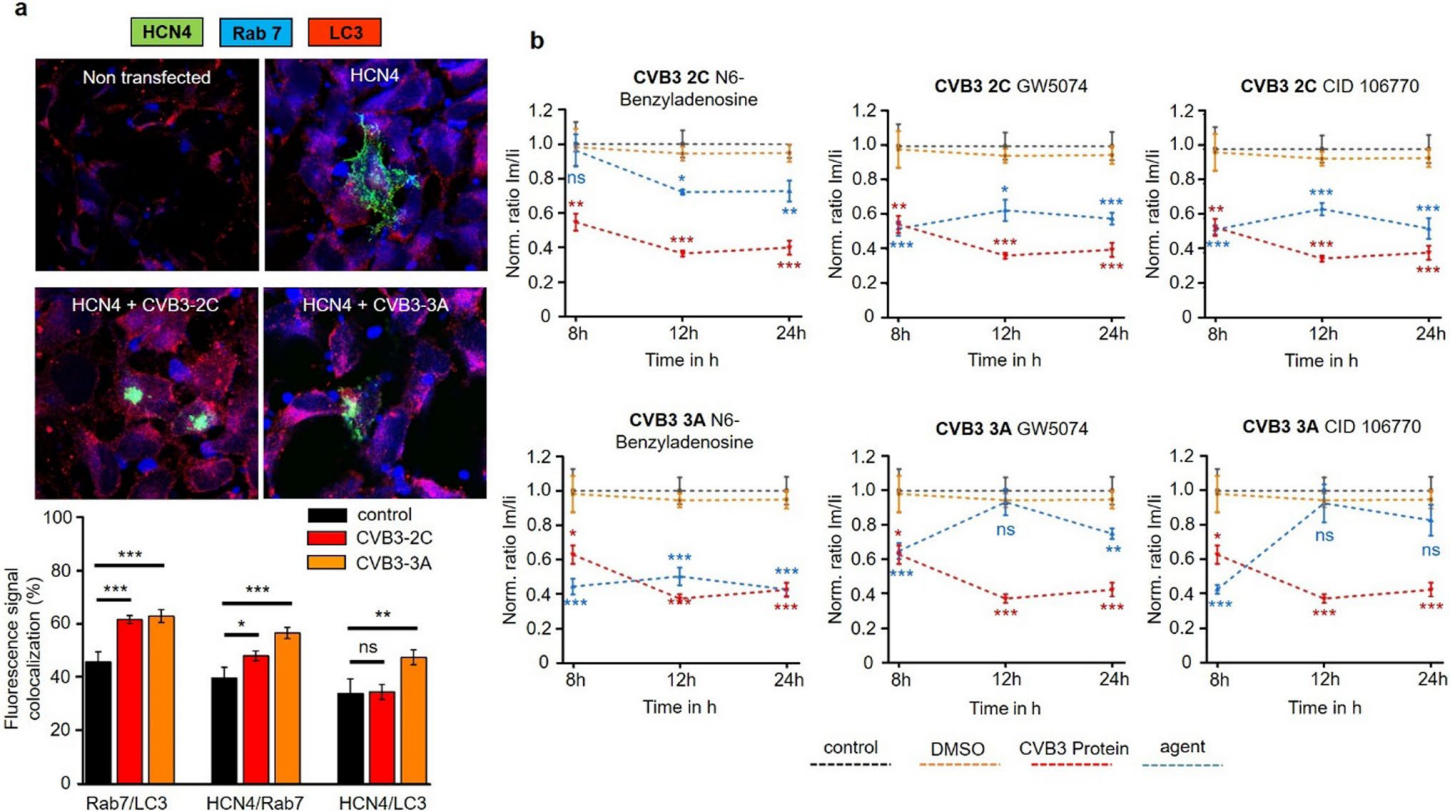
Interplay between HCN4, Rab7 and LC3 under coexpression of CVB3-2C or CVB3-3A. **a** Colocalization of HCN4, Rab7 and LC3 in HeLa cells only under HCN4 expression (*n* = 15) or under the coexpression of either CVB3-2C (*n* = 17) or CVB3-3A (*n* = 21). The HeLa cells were fixed and immunostained 12 h after transfection and colocalization of HCN4, Rab7 and LC3 was analyzed with JAcoP in ImageJ (*p < 0.05; **p < 0.01; ***p < 0.001). **b** Pharmacological treatment of CVB3-2C- or CVB3-3A-expressing HeLa cells for the potential reversion of the malignant, cytoplasmatic HCN4 accumulation. The treatment with either N6-Benzyladenosine, GW5074 or CID 106770 was done for 8 h, 12 h or 24 h after transfection with a CVB3-2C- or CVB3-3A-construct (*n* = 10). The cells were imaged and membranous (Im) versus cytoplasmatic (Ii) HCN4-GFP intensity was compared (*p < 0.05; **p < 0.01; ***p < 0.001)

Autophagy, the internal cellular mechanism for protein recycling, is known to be manipulated by enteroviruses like CVB3 for the enhanced generation of autophagosomes, which are abused as viral replication sites. Furthermore, the Rab7 GTPase is involved in intracellular autophagosome transport. This transport may be affected by CVB3 which may lead to the observed autophagosome and HCN4 accumulation in the cytoplasm. To investigate this hypothesis, we transfected HeLa cells with HCN4-eGFP alone or co-transfected with either CVB3-2C-dsRed or CVB3-3A-dsRed (Fig. 5c). The cells were then fixed and co-stained against Rab7 and the autophagosome marker LC3, because Rab7 is known to colocalize with autophagosomes and executes autophagosome transport within the cell. Colocalization assessment was performed to check for a potential colocalization enhancement of HCN4 with Rab7 and/or LC3. Cells transfected with CVB3-2C showed a general colocalization increase of Rab7 and LC3, which indicates autophagy induction in presence of CVB3-2C. Interestingly, HCN4 did not show increased colocalization with LC3. However, HCN4 and Rab7 showed a significant colocalization in presence of CVB3-2C. CVB3-3A led to an increased co-localization of Rab7 and LC3 as well. In addition, HCN4 was found to be strongly co-localized with Rab7 and LC3. This finding argues for an autophagy-coupled effect of CVB3-3A involved in relocalization of HCN4 (Fig. 7a). As CVB3-induced autophagy with Rab7 involvement was identified as one possible explanation for HCN4 relocalization in human cells, we addressed the question if inhibition of either CVB3-2C, CVB3-3A or Rab7 could at least partially reverse the altered HCN4 localization in heterologous expression. Therefore, HeLa cells were transfected with HCN4-eGFP alone or co-transfected with either CVB3-2C-dsRed or CVB3-3A-dsRed and treated with the CVB3-2C-inhibitor N6-Benzyladenosine, the CVB3-3A-inhibitor GW5074 or the Rab7-inhibitor CID 106770 for 8 h, 12 h or 24 h. The fluorescence intensity of the HCN4-eGFP signal was monitored for the membrane and the cytoplasmatic fraction via LASX and ratiometrically correlated. CVB3-2C-expressing HeLa cells showed no recovery in HCN4 distribution after 8 h of GW5074 or CID 106770 treatment. After 12 h and 24 h of treatment the HCN4 distribution slightly changed towards a more cytoplasmatic localization, but the effect was small and still many cytoplasmatic HCN4 aggregates were observed. Specific inhibition of CVB3-2C with N6-Benzyladenosine preserved HCN4 distribution comparable to the control after 8 h of treatment. After 12 h and 24 h of N6-Benzyladenosine treatment, the HCN4 distribution was still mainly membranous but cytoplasmatic HCN4 aggregates were observed as well, arguing for a transient effectivity of N6-Benzyladenosine against CVB3-2C. CVB3-3A-expressing HeLa cells showed no recovery effect after 8 h, 12 h or 24 h when treated with N6-Benzyladenosine at all. However, treatment with GW5074 or CID 106770 led to an almost complete recovery and thus to a normal HCN4 distribution after 12 h and 24 h of treatment, resembling a delayed treatment effect of both agents. Still, after 24 h of treatment, GW5074 starts to lose effectivity indicated by an increased fraction of cytoplasmatic HCN4 compared to cells treated with CID 106770. After 8 h of treatment, no recovery effect of GW5074 or CID 106770 was observed (Fig. 7b). In sum, these results support the hypothesis that enhanced Rab7-mediated autophagosomal transport is responsible for HCN4 channel redistribution in hiPSC-derived pacemaker-like cells and that pharmacological treatment with either Rab7 or CVB3-3A inhibitors could help to reduce the occurrence of cardiac disturbances during VMC.

## Discussion

The investigation of viral effects on the cardiac system was long-time underrepresented and pathogen–host–cell interaction was insufficiently studied. While the SAN with its containing pacemaker cells represents a small cytological area that is difficult to observe in the mammalian heart, viral infections of the SAN are only weakly understood by biomedical science. An anatomical study from 1992 showed structural changes in the SAN of C3H/He mice infected with CVB3. Cell degradation, accumulation of intracellular vacuoles and migrating macrophages indicated an inflammatory response to the virus [34]. Functional alterations or physiological consequences underlying these changes remained unresolved. It is known, that enteroviral infections of the cardiac system potentially lead to localized loss of functions in the working myocardium and to arrhythmicity under stress situations [32]. The arrhythmic alterations of the heart cannot be solely explained by local infection foci of the working myocardium in most cases. The development of directed differentiation protocols for the generation of hiPSC-derived pacemaker-like cells offer novel avenues to observe viral infections in detail [30].

**Fig. 8:**
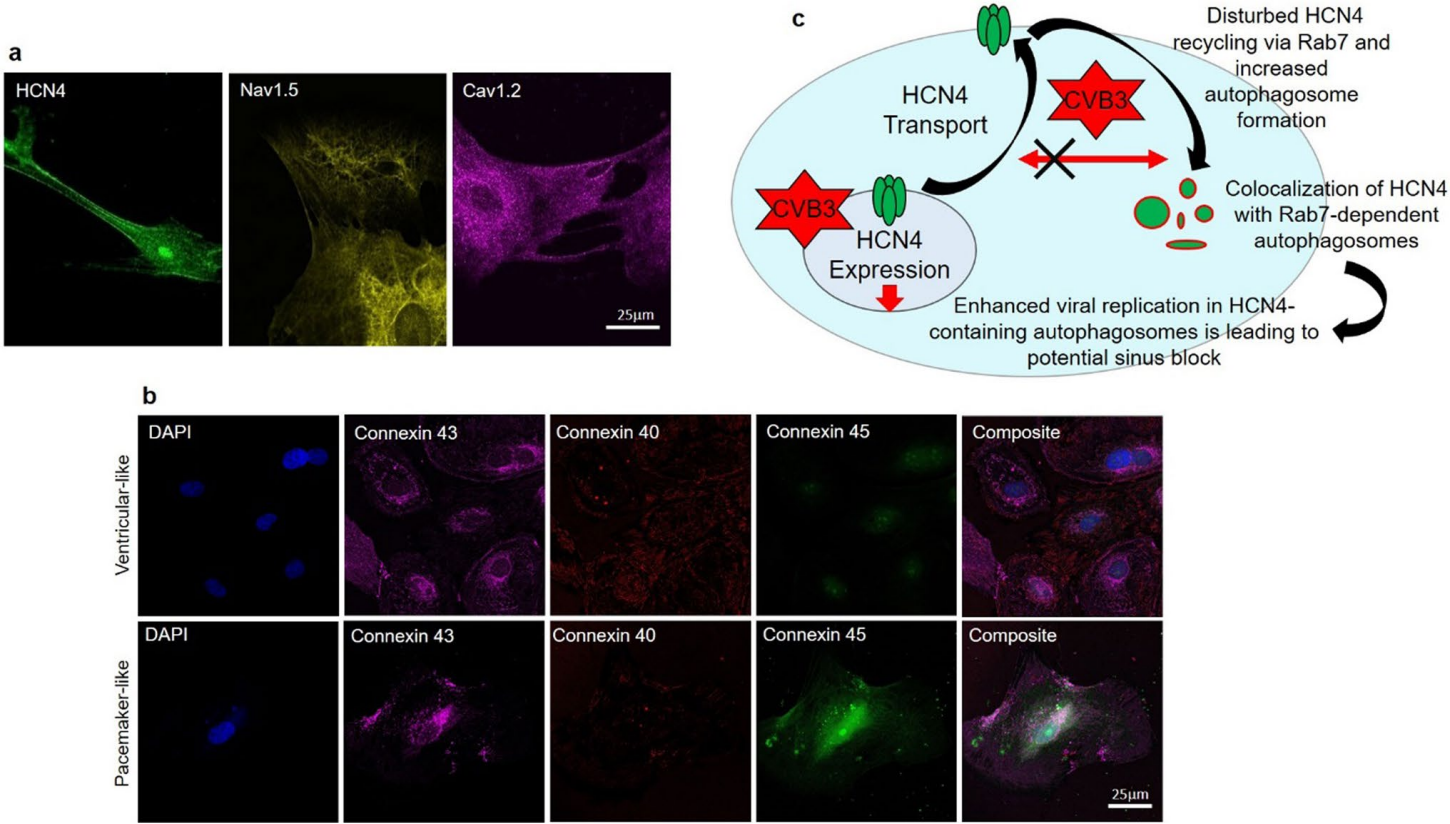
**a** Differentiated hiPSC-derived pacemaker cells express the SAN channel HCN4 and the cardiac channels Nav1.5 and Cav1.2. **b** Immunofluorescence staining of hiPSC-derived ventricular-like cells and hiPSC-derived pacemaker-like cells against connexin 43 (magenta), connexin 40 (red) and connexin 45 (green). Single channel pictures. **c** Schematic interaction between CVB3 and hiPSC-derived pacemaker cells concerning HCN4 transport and function as it was observed in this study. Not only is CVB3 a player in general HCN4 expression but mainly disturbs HCN4 recycling via Rab7-dependent autophagy combined with elevated autophagosome formation, leading to the loss of HCN4 function

The newly generated hiPSC line SFS.1CVB3∆VP0, which was used in this study, enables controlled CVB3 expression (Fig. 1a). Being pluripotent, these cells can be differentiated into several cardiac subtypes like ventricular-like and pacemaker-like cells differing in appearance and in the expression of specific connexins (connexin 43, connexin 40 and connexin 45). Connexin 45 thereby marks specifically for human SAN tissue and is mainly expressed in hiPSC-derived pacemaker-like cells (Fig. 1b, c) [38]. The observed connexin expression pattern obtained by FACS analysis was further investigated via immunofluorescent antibody staining of connexins 43, 40 and 45. As connexin 43 and 40 was clearly observed in both, hiPSC-derived ventricular-like cells and pacemaker-like cells, SAN-specific connexin 45 was only observed in hiPSC-derived pacemaker-like cells. This finding is in line with SAN-like specification of the hiPSC-derived pacemaker-like cells. Furthermore, it was clearly visible that connexin 40 signals overall appeared weaker in hiPSC-derived pacemaker cells compared to ventricular-like cells. The localization of connexin 43 was found to be mainly cytoplasmatic in hiPSC-derived pacemaker-like cells, whereas it was clearly located within the membranes of hiPSC-derived ventricular-like cells (Figs. 1d; 8b). This might indicate that connexin 43 is expressed in hiPSC-derived pacemaker-like cells, but might not fulfill connexon formation within the plasma membrane of neighboring cells. Anyhow, this observation has still to be evaluated in further detail. The characterization of the connexin expression and localization in both hiPSC-derived cell types indicates SAN-like specification of hiPSC-derived pacemaker-like cells as SAN-specific connexin 45 expression and membrane localization is solely observed in pacemaker-like cells (Figs. 1d; 8b). The increased contraction speed of hiPSC-derived pacemaker-like cells compared to hiPSC-derived ventricular-like cells further indicates SAN-like specification of the pacemaker-like cells (Fig. 1e; Video 1, 2). The expression of the cardiac ion channels Nav1.5 and Cav1.2 indicates a strong cardiac specification. Additional expression of the pacemaker channel HCN4 further qualifies the differentiated cells as model system for viral infections in the human SAN (Fig. 8a). Investigation of the ion channel distribution via immunofluorescence staining revealed several changes in HCN4 localization in CVB3-expressing pacemaker cells, in which the basic HCN4 membrane fraction was shifted towards an increased cytoplasmatic localization (Fig. 2a). As the localization of Nav1.5 and Cav1.2 was not significantly altered under CVB3 expression, the enteroviral effect seems specific to HCN4 (Fig. 2a, b). Furthermore, fluorescence intensity analysis exerts downregulation of HCN4 expression, while Nav1.5 and Cav1.2 remained unchanged (Fig. 2c). A reduced HCN4 function due to reduced expression and removal from the plasmamembrane to the cytoplasm can be expected to cause bradycardia, severe arrhythmic phenotypes and even SAN arrest in patients comparable to the sick sinus syndrome with genetically caused HCN4 defect due to insufficient pacemaker activity [1, 19]. As HCN4 is not only located in the SAN but also in other parts of the cardiac conduction system like the atrioventricular node and the His bundle, functional defects may be present in these compartments as well [40]. This might enhance the likelihood of cardiac block in patients, even if the SAN might not be directly infected by enteroviruses, but functional compartments downstream. To investigate, if the HCN4 re-distribution has any potential negative side effects in hiPSC-derived pacemaker-like cells, MEA recordings were performed to characterize the cells´ electrical activity. During pacemaker differentiation, the pacemaker-like cells´ membrane potentials appear immature in young age and are probably representing calcium fluxes through the membrane, as also reported in cardiac differentiations in other studies [41]. These immature pacemaker-like cells show a relatively fast signal generation rate and do not respond to pharmacological block of HCN4 with ivabradine (Fig. 3a, d). After a sufficient maturation time, a total of 6 weeks, the cells show a lowered autonomous rate and the generated signals show defined sodium-dependent depolarization and potassium-dependent repolarization. Furthermore, the matured pacemaker-like cells respond to ivabradine treatment with lowered activity and after 5 min with a complete ivabradine-induced block. Summarizing, maturation of the pacemaker-like cells switched the autonomous activity from calcium-dependent to a HCN4-driven mechanism, whose blockage with ivabradine was not compensated for another ion channel in the system (Fig. 3b, d). In mammal SAN tissue, one would expect similar effects, especially when it comes to pharmacological HCN4 inhibition [4]. The expression of CVB3 changed the autonomous rate to a similar level as observed in immature pacemaker-like cells. Anyhow, a detailed view on single extracellular signals suggests preserved sodium-dependent depolarization and potassium-dependent repolarization (Fig. 3c, d). It is to mention, that the potassium-dependent repolarization delayed after CVB3 expression, which is in line with previous studies suggesting a reduction in the rapid delayed rectifier K^+^ current (*I*_Kr_) [32]. The pharmacological inhibition of HCN4 in CVB3-expressing pacemaker-like cells had no effect on overall signal generation suggesting that HCN4 is no more the signal generator in the cells. As sodium and potassium potentials are preserved, a specific mechanism involving HCN4 modulation by CVB3 can be proposed as the reason for CVB3s´ effect on pacemaking. Autonomous activity independent of HCN4 function may induce spontaneous depolarisation in CVB3-expressing pacemaker-like aggregates (Fig. 3c, d). Accordingly, other ionic currents contribute to a “voltage-clock” that closely interacts with rhythmic ryanodine receptor-mediated intracellular calcium release (“calcium-clock”) [17]. Elevated depolarization due to I_Kr_ blockade, which would keep the cells closer to calcium channel activation may be another possible explanation for the observed spontaneous activity of CVB3-expressing cells [37]. To verify the change in HCN4 activity under the presence of CVB3, TEVC measurements on X*enopus laevis* oocytes were performed. The activity of HCN4 decreased tremendously when co-expressed with CVB3 (Fig. 4a). The analysis of the normalized currents indicates a highly significant reduction in overall HCN4 activity with *p* < 0.001 and tail current analysis supports this observation (Fig. 4b, c). The expression of single CVB3 proteins 2A-3D in *Xenopus laevis* oocytes identified the viral non-structural proteins CVB3-2C and CVB3-3A as potential modulators of HCN4 function (Fig. 5a, b). The identification of CVB3-2C and CVB3-3A as main causes for HCN4 loss-of-function led us to the question of which cellular mechanisms are manipulated by CVB3 resulting in the observed HCN4 displacement. As CVB3-3A is known to induce autophagy in CVB3-infected cells and as CVB3-2C was reported to mediate viral replication in autophagosomes, we proposed autophagy as one possible mechanism to explain HCN4 dysregulation in our system. The expression of CVB3 in hiPSC-derived pacemaker-like cells resulted in a general increase of autophagosome formation, indicated by the fluorescence signal increase of the autophagosomal marker proteins LC3, Beclin-1 and p62 (Fig. 6a, b.). Furthermore, the colocalization analysis of HCN4 with either autophagosomal LC3, Beclin-1 or p62 clearly showed an increase in colocalization when CVB3 was expressed (Fig. 6c.). These findings clearly point towards a prominent role of autophagy in HCN4 dysregulation.

As cytoplasmatic HCN4 localization and autophagy were found enhanced in CVB3-expressing cells, it raised the hypothesis that aberrant vesicular trafficking might be involved in the observed cytoplasmatic accumulation. As the cardiac Kv7.1 has previously been shown to be increasingly transported to the cell membrane via enhanced SGK1 and Rab11 activation during CVB3-2A expression, a viral disturbance of another Rab-dependent transport pathway for HCN4 might explain decreased membrane insertion and cytoplasmatic accumulation [32]. As the proteins CVB3-2C and CVB3-3A are strongly associated with the formation of the viral replication complex of enteroviruses including CVB3, the accumulation of HCN4 in cytoplasmatic aggregates and the induction of autophagosomes could originate from a similar mechanism [16, 28, 31, 33]. Autophagosomes are known to be used by CVB3 and other enteroviruses for viral replication, whereas the transport of those autophagosomes is directed via Rab7-dependent transport [7, 18, 22]. As CVB3 disturbs the native autophagy of the cell and induces the enhanced formation of autophagosomes in the cytoplasm for viral replication, this might result in a disturbed HCN4 degradation and recycling leading to the observed cytoplasmatic HCN4 aggregates. The co-localization of the autophagosome marker LC3 with Rab7 and HCN4 in CVB3-3A-expressing HeLa cells strongly supports this hypothesis. Interestingly, the colocalization of LC3, Rab7 and HCN4 was not observed in CVB3-2C-expressing HeLa cells, while LC3 and Rab7 colocalization was still observed (Fig. 7a). Treatment of HCN4- and CVB3-3A-expressing HeLa cells with the CVB3-3A inhibitor GW5074 or with the Rab7 inhibitor CID 106770 led to the recovery from the cytoplasmatic HCN4 accumulation into a healthy appearing phenotype, indicating that indeed malfunctioning Rab7-directed autophagosome transport is involved in the disturbed, cytoplasmatic HCN4 accumulation in CVB3-3A-expressing cells. As CVB3-2C-expressing cells did not respond to GW5074 or CID 106770, the observed accumulation in presence of CVB3-2C must be based on a different mechanism, which still has to be evaluated. Anyhow, as treatment with the CVB3-2C inhibitor N6-Benzyladenosine showed a recovery effect towards wildtype HCN4 distribution after 8 h, the cytoplasmatic HCN4 accumulation could be transiently counteracted, however without identifying the exact mechanism (Fig. 7b). As Rab7-driven autophagy is induced by CVB3-2C in a similar way as under CVB3-3A expression without linked HCN4 colocalization, the mechanism for CVB3-2C-dependent HCN4 redistribution must have a different origin from Rab7-dependent autophagy. Rab7 was found to be colocalized with HCN4 when CVB3-2C was expressed, leading to the assumption that dysregulated Rab7 transport may provide a partial explanation for cytoplasmatic HCN4 accumulation. Still, whether vesicular trafficking or another cellular transport mechanism is manipulated by CVB3-2C leading to the formation of intracellular HCN4 accumulates has to be established.

Summarizing, CVB3 expression inhibits human cardiac pacemaker function by reducing the pace maker channel plasma membrane density. This process depends on RAB7 and the CVB3 effect can be corrected by pharmacological intervention of endocytic vesicle trafficking (Fig. 8c). Potentially, RAB7 inhibition may present a pharmacological approach to treat bradycardia and prevent cardiac arrest in CVB3 infected patients to reduce mortality.

### Supplementary Information

Below is the link to the electronic supplementary material.

Supplementary file1 (AVI 29912 kb)

Supplementary file2 (AVI 30500 kb)

## Data Availability

The datasets generated during and/or analysed during the current study are available from the corresponding author on reasonable request.
